# The Effectiveness of Technology-Based Cardiopulmonary Resuscitation Training on the Skills and Knowledge of Adolescents: Systematic Review and Meta-analysis

**DOI:** 10.2196/36423

**Published:** 2022-12-15

**Authors:** Xiu Ming Amanda Lim, Wenxin Ariel Liao, Wenru Wang, Betsy Seah

**Affiliations:** 1 Woodlands Health Singapore Singapore; 2 Alice Lee Centre for Nursing Studies Yong Loo Lin School of Medicine National University of Singapore Singapore Singapore

**Keywords:** cardiac arrest, education, methods, first responders, resuscitation training, effectiveness, adolescents, schoolchildren

## Abstract

**Background:**

Cardiopulmonary resuscitation (CPR) training for adolescents is a prominent strategy to increase the number of community first responders who can recognize cardiac arrest and initiate CPR. More schools are adopting technology-based CPR training modalities to reduce class time and reliance on instructor availability and increase their capacity for wider training dissemination. However, it remains unclear whether these technology-based modalities are comparable with standard training.

**Objective:**

This study aimed to systematically review and perform meta-analyses to evaluate the effectiveness of technology-based CPR training on adolescents’ CPR skills and knowledge.

**Methods:**

Searches were conducted in PubMed, Embase, Cochrane Library, Ovid MEDLINE, CINAHL, PsycINFO, Education Resources Information Center, ProQuest Dissertations and Theses Global, and Scopus from inception to June 25, 2021. Eligible randomized controlled trials (RCTs) compared technology-based training with standard training for adolescents aged 12 to 18 years. Studies were appraised using the Cochrane risk-of-bias tool. Random-effects meta-analyses were performed using Review Manager (The Cochrane Collaboration). Subgroup analyses were conducted to explore sources of heterogeneity. Overall certainty of evidence was appraised using the Grading of Recommendations Assessment, Development, and Evaluation approach.

**Results:**

Seventeen RCTs involving 5578 adolescents were included. Most of the studies had unclear risks of selection bias (9/17, 53%) and high risks of performance bias (16/17, 94%). Interventions that included instructor guidance increased the likelihood of adolescents checking the responsiveness of the person experiencing cardiac arrest (risk ratio 1.39, 95% CI 1.19-1.63) and calling the emergency medical services (risk ratio 1.11, 95% CI 1.00-1.24). Self-directed technology-based CPR training without instructor guidance was associated with poorer overall skill performance (Cohen *d*=–0.74, 95% CI –1.02 to –0.45). Training without hands-on practice increased mean compression rates (mean difference 9.38, 95% CI 5.75-13.01), whereas real-time feedback potentially yielded slower compression rates. Instructor-guided training with hands-on practice (Cohen *d*=0.45, 95% CI 0.13-0.78) and the use of computer programs or mobile apps (Cohen *d*=0.62, 95% CI 0.37-0.86) improved knowledge scores. However, certainty of evidence was very low.

**Conclusions:**

Instructor-guided technology-based CPR training that includes hands-on practice and real-time feedback is noninferior to standard training in CPR skills and knowledge among adolescents. Our findings supported the use of technology-based components such as videos, computer programs, or mobile apps for self-directed theoretical instruction. However, instructor guidance, hands-on practice, and real-time feedback are still necessary components of training to achieve better learning outcomes for adolescents. Such a blended learning approach may reduce class time and reliance on instructor availability. Because of the high heterogeneity of the studies reviewed, the findings from this study should be interpreted with caution. More high-quality RCTs with large sample sizes and follow-up data are needed. Finally, technology-based training can be considered a routine refresher training modality in schools for future research.

## Introduction

### Background

Out-of-hospital cardiac arrests (OHCAs) are associated with poor survival and neurological outcomes [1]. Prognoses are improved when bystanders promptly recognize cardiac arrest and initiate cardiopulmonary resuscitation (CPR) [2]. Of note, many laypeople lack the ability to identify OHCA or act appropriately. The American Heart Association and European Resuscitation Council advocate compulsory annual CPR training for individuals aged ≥12 years [3]. Generally, students receiving formal education (ie, middle school and high school students) are aged between 12 and 18 years. Introducing CPR training in schools can equip a large proportion of the country’s population with fundamental lifesaving knowledge and skills. The early introduction of these lifesaving skills during one’s developmental years not only promotes altruism but also increases one’s willingness to help people experiencing OHCA [4]. Such school-based education sessions can prepare both students and teachers in responding to cardiac arrest incidents within schools and the community at large. Moreover, regular refresher training can be arranged easily in schools because most children attend formal education [5]. However, the lack of stringent guidelines gives schools full autonomy to conduct diverse training modalities, some of which are yet to be supported by empirical evidence [6].

Standard CPR training involves didactic face-to-face lessons, skill demonstrations by qualified instructors, and hands-on practice on manikins in small groups [7]. Although this modality has been regarded as the gold standard, there are often not enough qualified instructors for large-scale implementation in schools. Such training requires numerous manikins and equipment, which are costly [8]. Furthermore, standard training consumes substantial class time and impedes adoption by schools [9].

### Technology-Based CPR Training

International resuscitation guidelines suggest the incorporation of technology into CPR training as alternatives to standard training [10,11]. Particularly in the age of the COVID-19 pandemic, technology-based CPR training has become increasingly prominent. These interventions are facilitated by digital technology, including instructional videos, web-based learning, computer programs, mobile apps, or advanced manikin software [12]. Many of them use self-directed learning to decrease reliance on the availability of qualified instructors [13]. Technology-based CPR training may also be cost-effective because fewer resources are required [14]. Finally, training duration is kept minimal, which complements hectic school curricula [15]. Hence, there is a tremendous potential for the proliferation of technology-based CPR training among adolescents.

Two systematic reviews were conducted on CPR training modalities for adolescents. Plant and Taylor [16] concluded that all modalities, including technology-based training, improved knowledge and skills. Reveruzzi et al [17] added that qualified instructors, videos, and hands-on practice improved training outcomes. Both reviews had broad eligibility criteria and no restrictions on study design. This contributed to heterogenous results that prevented pooling of training effects using meta-analyses. Other systematic reviews involving technology-based training focused on health care students, health care professionals, and adult laypeople [18-20]. The conclusions from these reviews cannot be generalized to adolescents because different teaching approaches might be necessary to cater to learners of different age groups [10]. This review used meta-analysis to synthesize evidence on the effectiveness of technology-based CPR training compared with standard training in improving the skills and knowledge of adolescents aged 12 to 18 years.

## Methods

This study adhered to the PRISMA (Preferred Reporting Items for Systematic Reviews and Meta-Analyses) guidelines [21].

### Search Strategy

Searches were conducted in PubMed, Embase, Cochrane Library, Ovid MEDLINE, CINAHL, PsycINFO, Education Resources Information Center, ProQuest Dissertations and Theses Global, and Scopus from inception to June 25, 2021. The search terms included adolescent*, schoolchild*, student*, cardiopulmonary resuscitation, basic life support, train*, and teach*. Synonyms were combined with the Boolean operator *OR*. Population and intervention concepts were then combined, such as *adolescents* AND CPR AND *training.* Refer to Multimedia Appendix 1 for the database search strategies. ClinicalTrials.gov and the World Health Organization International Clinical Trials Registry Platform were searched for ongoing and unpublished trials. Hand searching of the *Resuscitation* journal was performed for articles published between January 2000 and June 2021. The reference lists of relevant trials and systematic reviews were screened to identify additional studies.

### Inclusion Criteria

Randomized controlled trials (RCTs) were included if they met the following criteria: (1) participants were adolescents aged 12 to 18 years; (2) participants received CPR training that included technology-based components such as videos, web-based learning, computer programs, mobile apps, or manikin software with real-time feedback; (3) technology-based CPR training was compared with standard CPR training (without the technology-based intervention component); and (4) the RCTs reported CPR skills or knowledge. CPR skills are defined as the ability to perform CPR techniques objectively measured via manikin software or as evaluated by instructors. Theoretical knowledge scores are measured by self-reported instruments, including multi-item questionnaires or multiple-choice–question tests (Table 1).

**Table 1:** Inclusion and exclusion criteria.

Variable					Inclusion criteria			Exclusion criteria					
**Study characteristics**													
	Study design			RCTs^a^ and cluster RCTs					Nonrandomized studies, observational studies, qualitative studies, and reviews				
	Publication type		Full-text journal publications, conference proceedings, and unpublished dissertations or theses				Editorials and letters						
	Publication year		No limit				N/A^b^						
	Language		English only				Languages other than English						
**PICO^c^ framework**													
	Population			Schoolchildren aged 12 to 18 years					Schoolchildren with physical disabilities that may affect their ability to perform CPR^d^ (eg, those who are blind, deaf, or have a speech disability)				
	Intervention		CPR training with technology-based components, including videos, computer programs, mobile apps, and real-time audiovisual feedback				CPR training with popular songs only						
	Comparison		Standard resuscitation training without technology-based component				N/A						
	**Outcomes**												
		Skill performance				Overall performance (cumulative score from skills checklist); components of cardiopulmonary resuscitation, including checking responsiveness, checking the airway and breathing, calling the EMS^e^, compression depth, compression rate, correct hand position, correct compression:ventilation ratio, total compressions, correct ventilation, AED^f^ pad placement, and use of AED				N/A			
		Knowledge				Theoretical knowledge scores				N/A			

^a^RCT: randomized controlled trial.

^b^N/A: not applicable.

^c^PICO: Population, Intervention, Comparison, and Outcomes.

^d^CPR: cardiopulmonary resuscitation.

^e^EMS: emergency medical services.

^f^AED: automated external defibrillator.

### Study Selection

All retrieved records were imported into EndNote X9 (Clarivate) for deduplication. Titles and abstracts of records were screened by 2 independent reviewers (AL and WX) for relevance. After removing irrelevant records, full texts of potential studies were independently assessed for eligibility. Discrepancies were resolved through discussion with a third reviewer (BS).

### Data Extraction

AL and WX collected data independently using a standardized data extraction form. Extracted data included publication year, country, study design, setting, participants, sample size, interventions, comparators, outcome measures, and instruments. Posttraining and retention data were extracted, with retention defined as at least 4 weeks after training. Indicators of trial quality were also extracted; for example, attrition rate, intention to treat, and trial registration. Results of studies reported in >1 publication were extracted as 1 study. Authors were contacted when data were incomplete or unclear. Discrepancies in extracted data were resolved through discussion with BS.

### Quality Appraisal

AL and WX performed quality appraisal independently for all included studies. Discrepancies were resolved through discussion with BS. The Cochrane risk-of-bias tool was used to appraise studies for risks of bias [22]. The Grading of Recommendations Assessment, Development, and Evaluation approach was used to appraise certainty of evidence for the main outcomes [23]. Ratings were categorized as high, moderate, low, or very low.

### Data Analysis

Meta-analyses were performed with Review Manager (The Cochrane Collaboration) and presented as forest plots where appropriate. The Mantel-Haenszel approach and risk ratio (RR) were selected for dichotomous outcomes, whereas the inverse-variance approach pooled mean differences (MDs) for continuous outcomes. Continuous outcomes measured using different scales were presented as standardized MDs or Cohen *d*. When mean and SD were not reported, values were estimated using median and IQR. Overall intervention effects were interpreted using the *Z* statistic, with level of significance set at *P*≤.05.

Heterogeneity was evaluated using the Cochran *Q* test and *I*^2^ statistic, with level of significance set at *P*≤.10. A random-effects model was used because of variation in training characteristics [24]; for instance, the studies used different modes of technology-based instruction as well as different types of CPR instructors such as health care professionals, schoolteachers, or medical students, which may affect effect sizes across the studies. For meta-analyses with significant heterogeneity and with at least 6 comparisons, sensitivity or subgroup analyses were performed [25]. The subgroup analyses explored potential effect modifiers, including instructor guidance, hands-on practice, and training modalities. Sensitivity analyses were conducted when meta-analyses yielded significant heterogeneity that was attributable to an outlier study. Funnel plots were not performed because of the limited number of trials included in each meta-analysis. Where quantitative analysis could not be determined from the meta-analysis, findings were presented narratively.

### Ethics Approval

The preparation of this paper did not involve primary research or data collection involving human participants; therefore, no institutional review board examination or approval was required.

## Results

### Study Characteristics

**Figure 1:**
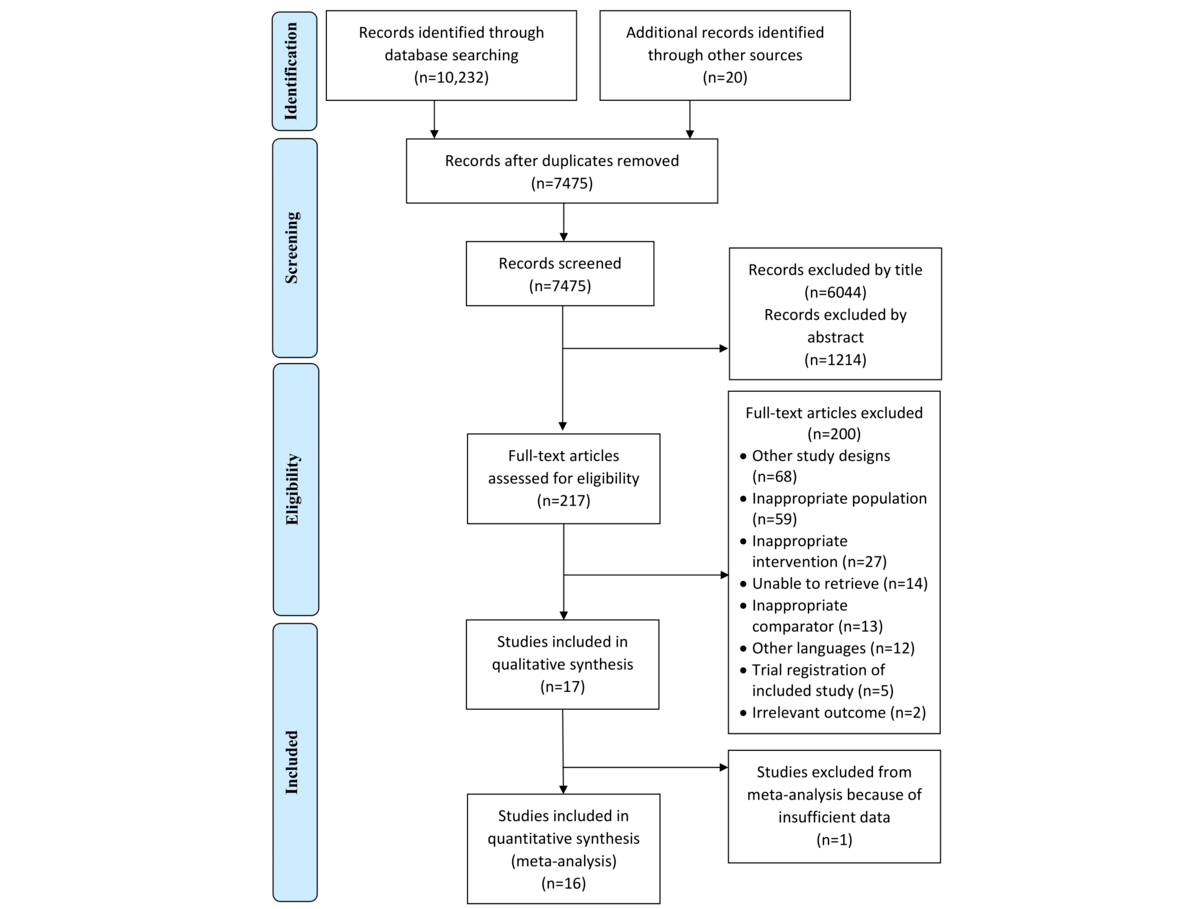
PRISMA (Preferred Reporting Items for Systematic Reviews and Meta-Analyses) flow diagram.

The search process is illustrated in Figure 1. Seventeen RCTs were included in this review. Table 2 summarizes the characteristics of the included studies. Of the 28 intervention arms included in this review, 4 (14%) [26-29] were excluded on account of irrelevance. Studies were conducted across 11 countries: Belgium, Canada, Iran, Italy, South Korea, the Netherlands, Spain, Sweden, Turkey, the United Kingdom, and the United States. A total of 5578 secondary school or high school students were recruited (sample sizes ranged from 62 to 1426). Of the 17 studies, 6 (35%) excluded students with prior or recent CPR training [26,27,30-33].

**Table 2:** Characteristics of the included studies.

Study authors, year; country	Study design	Sample size	Technology-based training	Standard training	Outcomes	Attrition (%)	ITT^a^; missing data management	Protocol; trial registration; funding
Beskind et al [28], 2016; United States	Three-arm cluster RCT^b,c^	I^d^: 58, C^e^: 54	Brief video	Standard instructor-led training	Skills	11.2	No; no	No; no; yes
Chamdawala et al [34], 2021; United States	Two-arm RCT	I: 110, C: 110	QCPR^f^ real-time visual feedback	Standard instructor-led training	Skills and knowledge	13.6	No; no	No; no; yes
Cortegiani et al [35], 2017; Italy	Two-arm RCT	I: 60, C: 65	QCPR real-time visual feedback	Standard instructor-led training	Skills	13.2	No; no	Yes; yes; no
Cuijpers et al [36], 2011; Netherlands	Three-arm RCT	I1: 33, I2: 34, C: 37	I1: 1 hour e-learning+1 hour instructor-led training, I2: 1 hour e-learning	Standard instructor-led training	Skills	NR^g^	NR; NR	NR; NR; NR
Doucet et al [37], 2019; Belgium	Two-arm RCT	I: 83, C: 82	StartnHart app	Standard instructor-led training	Skills	0	N/A^h^	No; no; no
Han et al [38], 2021; Korea	Two-arm RCT	I: 31, C: 31	e-Learning+videoconferencing	Standard instructor-led training	Skills	0	N/A	No; no; yes
Iserbyt et al [31], 2014; Belgium	Two-arm RCT	I: 59, C: 69	Video instruction	Static picture instruction	Skills	7.2	No; no	No; no; no
Marchiori et al [39], 2012; Spain	Two-arm cluster RCT	I: 187, C: 144	Video game	Standard instructor-led training	Knowledge	3.8	No; no	No; no; yes
Morrison et al [40], 2012; Canada	Two-arm RCT	I: 140, C: 124	CPR^i^ Anytime video self-instruction+instructor-led training for AED^j^	Standard instructor-led training	Skills	21	NR; NR	NR; NR; NR
Nord et al [41], 2017; Sweden	Two-arm cluster RCT	I: 645 or 208, C: 587 or 224	Web course+classroom-based instructor-facilitated training with app (static pictures) or video instruction	Classroom-based instructor-facilitated training with app (static pictures) or video instruction	Skills and knowledge	13.6	No; no	No; yes; yes
Norman [26], 1984; United States	Three-arm RCT^c^	I: 39, C: 39	Video instruction	Standard instructor-led training	Skills and knowledge	17.1	No; no	NR; NR; NR
Onan et al [42], 2019; Turkey	Three-arm cluster RCT	I1: 25, I2: 25, C: 25	I1: video instruction, I2: video instruction with real-time feedback	Standard instructor-led training (theory only)	Skills and knowledge	7.2	No; no	No; no; no
Otero-Agra et al [32], 2019; Spain	Four-arm cluster RCT	I1: 151, I2: 140, I3: 109, C: 89	I1: mandatory and graded team-based training with real-time feedback for competition, I2: mandatory and graded individual training with real-time feedback, I3: individual training with real-time feedback	Standard instructor-led training	Skills	0	N/A	No; no; no
Reder et al [29], 2006; United States	Four-arm cluster RCT^c^	I1: 213, I2: 170, C: 206	I1: interactive computer session, I2: interactive computer session with practice	Standard instructor-led training	Skills and knowledge	22.8	No; no	No; no; NR
Rezaei et al [33], 2013; Iran	Two-arm cluster RCT	I: 42, C: 42	Prerecorded video demonstration	Standard instructor-led training	Skills and knowledge	0	N/A	No; no; yes
Van Raemdonck et al [27], 2014; Belgium	Four-arm RCT^c^	I1: 44, I2: 42, C: 43	I1: video instruction, I2: video instruction with low-cost manikin	Standard instructor-led training	Skills	66.3	No; no	No; no; yes
Yeung et al [30], 2017; United Kingdom	Three-arm cluster RCT	I1: 21, I2: 24, C: 19	I1: Lifesaver app, I2: Lifesaver app+standard instructor-led training	Standard instructor-led training	Skills	21	No; no	No; yes; yes

^a^ITT: intention to treat.

^b^RCT: randomized controlled trial.

^c^One comparison arm was not included in this review because of an irrelevant comparator.

^d^I: intervention.

^e^C: control.

^f^QCPR: quality cardiopulmonary resuscitation.

^g^NR: not reported.

^h^N/A: not applicable.

^i^CPR: cardiopulmonary resuscitation.

^j^AED: automated external defibrillator.

### Descriptions of Interventions and Comparators

All studies adhered to national or international resuscitation guidelines, except for the study by Rezaei et al [33], which did not mention them. Only the study by Iserbyt et al [31] used a multimedia learning theory to guide the intervention. Training was either self-directed [27-31,33,37,39] or instructor guided [26,29,30,33-36,38,40-42]. Trained schoolteachers or medical students served as instructors or facilitators in 29% (5/17) of the studies [29,31,33,41,42]. The interventions comprised video instruction [26-28,31,33,40,42], computer programs or mobile apps [29,30,36-39,41,42], or real-time feedback [32,34,35]. Of the 24 intervention arms, 4 (17%) omitted hands-on practice on manikins [28-30,33]. All studies reported up to 2 training sessions over a span of 3 weeks, with each session lasting from 1.5 minutes to 4 hours. The length of follow-up ranged from 2 months [28,29] to 1 year [34].

The standard training used included face-to-face qualified-instructor–led demonstration, with hands-on practice on manikins. Other comparators included static pictures [31], classroom-based video instruction [31,34,38,41], or didactic teaching without hands-on practice [42]. Refer to Multimedia Appendix 2 for details.

### Quality Assessment

**Figure 2:**
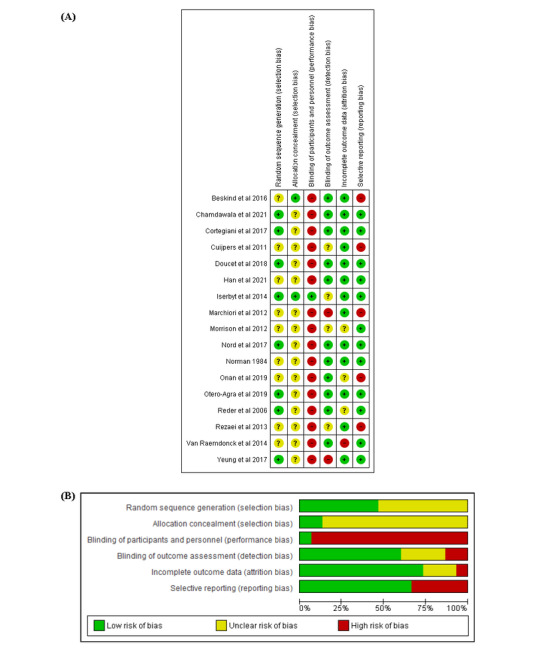
Risk-of-bias (A) summary and (B) graph.

Most (12/17, 71%) of the studies had overall moderate risk of bias (Figure 2). There was low risk of selection bias in 47% (8/17) of the RCTs because of adequate random sequence allocation and in 12% (2/17) because of allocation concealment. Because of the nature of CPR training, blinding of participants and personnel was not possible in all of the trials. Of the 17 RCTs, 7 (41%) had low risk of detection bias because of the blinding of outcome assessors and another 4 (24%) trials had all outcomes objectively measured through manikins, minimizing bias attributable to the lack of blinding. Of the 17 studies, 13 (76%) had low risk for attrition bias because of similar reasons for attrition across the groups or no missing data. Although only 12% (2/17) of the studies published protocols, 59% (10/17) reported all outcomes completely and were thus rated low risk for reporting bias. Certainty of evidence was appraised as *very low* for skills and knowledge using the Grading of Recommendations Assessment, Development, and Evaluation approach (Multimedia Appendix 3). Domains were downgraded because of high risks of bias, statistical and methodological heterogeneity, small sample sizes, and wide CIs.

### Effectiveness of Technology-Based Training on Overall Performance

#### Overview

Overall performance is the cumulative score from a skills checklist, with components presented in Table 3. The sole use of self-directed learning yielded poorer overall performance after the intervention. At 6 months, technology-based training potentially improved overall performance.

**Table 3:** Meta-analyses: cardiopulmonary resuscitation skill components.

Outcome and time point				Trials (N=16), n (%)		Arms (N=23), n (%)		Sample size (N)			Statistical approach			Effect estimate (95% CI)		Overall effect					*I*^2^ (%)
																*Z* statistic		*P* value			
**First link in chain of survival: early recognition and calling for help**																					
	**Checking responsiveness**																				
		After training	5 (31)		6 (26)		884		M-H^a^, random effects			1.16^b^ (0.90 to 1.50)			1.14		.25		88		
	**Checking airway**																				
		After training	5 (31)		6 (26)		1370			M-H, random effects			0.93^b^ (0.78 to 1.10)		0.85		.39		60		
		Retention	2 (13)		3 (13)		892			M-H, random effects			0.90^b^ (0.72 to 1.13)		0.89		.37		25		
	**Checking breathing**																				
		After training	4 (25)		5 (22)		719			M-H, random effects			1.18^b^ (0.92 to 1.50)		1.31		.19		68		
	**Calling EMS^c^**																				
		After training	6 (38)		7 (30)		996		M-H, random effects			1.10^b^ (0.92 to 1.31)			1.07		.28		81		
		Retention	2 (13)		2 (9)		511		M-H, random effects			1.01^b^ (0.92 to 1.10)			0.14		.89		0		
**Second link in chain of survival: early CPR^d^**																					
	**Overall compression quality (%)**																				
		After training	3 (19)		4 (17)		824			IV^e^, random effects			23.96^f^ (19.84 to 28.09)		11.40		*<.001^g^*		0		
	**Mean compression depth (mm)**																				
		After training	10 (63)		13 (57)		1619			IV, random effects			1.16^f^ (–2.49 to 4.82)		0.62		.53		95		
		Retention	6 (38)		8 (35)		1179			IV, random effects			0.73^f^ (–3.07 to 4.52)		0.38		.71		94		
	**Correct compression depth**																				
		After training	6 (38)		8 (35)		1447			M-H, random effects			1.04^b^ (0.90 to 1.21)		0.54		.59		43		
		Retention	2 (13)		3 (13)		528			M-H, random effects			0.76^b^ (0.59 to 0.97)		2.17		*.03*		0		
	**Mean compression rate (number of compressions per minute)**																				
		After training	11 (69)		15 (65)		2107			IV, random effects			–3.25^f^ (–7.57 to 1.07)		1.47		.14		88		
		Retention	6 (38)		8 (35)		1179			IV, random effects			–2.47^f^ (–7.48 to 2.54)		0.97		.33		85		
	**Correct compression rate**																				
		After training	5 (31)		7 (30)		601			M-H, random effects			0.89^b^ (0.75 to 1.07)		1.22		.22		38		
	**Correct hand position**																				
		After training	7 (44)		10 (43)		1617			M-H, random effects			0.93^b^ (0.84 to 1.03)		1.38		.17		44		
		Retention	3 (19)		5 (22)		1021			M-H, random effects			0.86^b^ (0.65 to 1.14)		1.06		.29		56		
	**Correct ventilation**																				
		After training	8 (50)		11 (48)		1680			M-H, random effects			0.86^b^ (0.67 to 1.10)		1.23		.22		69		
		Retention	3 (19)		5 (22)		1056			M-H, random effects			0.64^b^ (0.41 to 0.99)		2.00		*.05*		78		
	**Correct compression:ventilation ratio**																				
		After training	2 (13)		2 (9)		597			M-H, random effects			0.99^b^ (0.87 to 1.13)		0.15		.88		34		
	**Total compressions in 2 minutes**																				
		After training	2 (13)		3 (13)		614			IV, random effects			–22.84^f^ (–30.35 to –15.33)		5.96		*<.001*		0		
**Third link in chain of survival: early defibrillation**																					
	**Correct AED** ^h^ **pad placement**																				
		After training	2 (13)		3 (13)		729			M-H, random effects			0.94^b^ (0.86 to 1.02)		1.58		.11		54		
	**Correct use of AED**																				
		After training	2 (13)		3 (13)		729			M-H, random effects			0.98^b^ (0.94 to 1.01)		1.15		.25		68		

^a^M-H: Mantel-Haenszel.

^b^RR: risk ratio.

^c^EMS: emergency medical services.

^d^CPR: cardiopulmonary resuscitation.

^e^IV: inverse variance.

^f^MD: mean difference.

^g^Results of significance are presented in italics.

^h^AED: automated external defibrillator.

#### Posttraining Performance

Of the 16 RCTs included in the meta-analyses for posttraining performance, 6 (38%; arms: 8/23, 35%) involving 1121 students reported overall performance scores from skills checklists [30,36,37,40-42]. Meta-analysis revealed high heterogeneity (*I*^2^=89%; *P*<.001). Subgroup analyses revealed that self-directed learning yielded significantly poorer overall performance (Cohen *d*=–0.74, 95% CI –1.02 to –0.45; *P*<.001; Table 4).

**Table 4:** Subgroup analyses based on instructor guidance: overall performance scores.

Subgroup analyses		Comparisons (n)	Effect estimate (95% CI)	Subgroup effect		*I*^2^ (%)	Subgroup differences			
				*Z* statistic	*P* value		*I*^2^ (%)		*P* value	
	Self-directed learning	2	–0.74 (–1.02 to –0.45)	5.02	*<.001* ^a^	0		92.8		*<.001*
	Instructor-guided learning	6	0.28 (–0.17 to 0.73)	1.22	.22	88		N/A^b^		N/A

^a^Results of significance are presented in italics.

^b^N/A: not applicable.

#### Retention

Of the 16 RCTs, 3 (19%; arms: 4/23, 17%) involving 727 students reported overall performance at 6 months [30,40,41]. Only instructor-guided training involving a participative mobile app significantly improved performance retention [30]. Interventions that used video instruction, a supplementary web-based course, or self-directed learning with a participative mobile app yielded performance similar to that of standard training [30,40,41].

### Effectiveness of Technology-Based Training on CPR Skill Components

Meta-analyses performed for CPR skill components are summarized in Table 3.

#### Posttraining Performance

**Figure 3:**
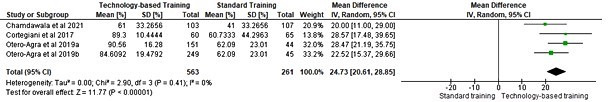
Meta-analysis: overall compression quality.

Of the 16 RCTs, 3 (19%; arms: 4/23, 17%) involving 824 students reported overall compression quality calculated by manikin software (Figure 3) [32,34,35]. Technology-based training significantly improved compression quality (MD 23.96, 95% CI 19.84-28.09; *P*<.001; Table 3). With significant heterogeneity reported for the other CPR components, sensitivity or subgroup analyses were performed for instructor guidance, training components, and training modalities (Table 5 and Multimedia Appendix 4). Sensitivity analyses of outlier studies did not yield statistically significant data.

**Table 5:** Subgroup analyses: cardiopulmonary resuscitation skill components after training and at retention.

Subgroup analyses					Comparisons (n)				Effect estimate (95% CI)			Subgroup effect									*I*^2^ (%)			Subgroup differences			
												*Z* statistic				*P* value							*I*^2^ (%)				*P* value
**Subgroup analyses based on instructor guidance**																											
	**Checking responsiveness (after training): 5 trials (6 arms)**																										
			Self-directed learning	3				1.07 (0.83 to 1.38)			0.50				.61				86			67				*.08^a^*	
			Instructor-guided learning	3				1.39 (1.19 to 1.63)			4.10				*<.001*				0			—^b^				—	
	**Checking airway (after training): 5 trials (6 arms)**																										
		Self-directed learning		4				0.84 (0.75 to 0.94)		3.05				*.002*				0						4.6	.31		
		Instructor-guided learning		2				1.02 (0.71 to 1.48)		0.13				.90				63						—	—		
	**Calling EMS^c^ or help (after training): 6 trials (7 arms)**																										
		Self-directed learning		4				1.10 (0.85 to 1.43)			0.72				.47				85			0				.93	
		Instructor-guided learning		3				1.11 (1.00 to 1.24)			2.01				*.04*				0			—				—	
	**Mean compression depth (after training): 10 trials (13 arms)**																										
		Self-directed learning				6			–3.16 (–8.17 to 1.85)			1.23				.22				93			83.9				*.01*
		Instructor-guided learning				7			3.94 (1.50 to 6.37)			3.17				*.002*				78			—				—
	**Correct hand position (after training): 7 trials (10 arms)**																										
		Self-directed learning		5				0.84 (0.71 to 0.99)			2.10				*.04*				48			64.1				*.09*	
		Instructor-guided learning		5				1.11 (0.83 to 1.47)			0.71				.48				74			—				—	
**Subgroup analyses based on hands-on practice**																											
	**Mean compression depth (after training): 10 trials (13 arms)**																										
		Hands-on practice		11				2.20 (0.08 to 4.32)			2.03		*.04*				79						70.6				*.07*
		Without practical training		2				–6.52 (–15.53 to 2.50)			1.42		.16				94						—				—
	**Mean compression rate (after training): 11 trials (15 arms)**																										
		Hands-on practice					13		–5.47 (–9.26 to –1.68)			2.83				*.005*				81			96.7				*<.001*
		Without practical training					2		9.38 (5.75 to 13.01)			5.07				*<.001*				0			—				—
	**Mean compression rate (retention): 6 trials (8 arms)**																										
		Hands-on practice		6				–3.88 (–9.79 to 2.03)			1.29				.20				86			62.1				*.10*	
		Without practical training		2				1.80 (–1.67 to 5.27)			1.02				.31				0			—				—	
**Subgroup analyses based on training modalities**																											
	**Correct hand position (after training): 7 trials (10 arms)**																										
		Video instruction		4				0.78 (0.61 to 1.00)			1.92				*.05*				0			9.3				.33	
		Computer program or mobile app		5				0.99 (0.82 to 1.18)			0.16				.87				66			—				—	
		Real-time feedback only		1				0.93 (0.84 to 1.02)			1.53				.13				N/A^d^			—				—	

^a^Results of significance are presented in italics.

^b^Not available.

^c^EMS: emergency medical services.

^d^N/A: not applicable.

#### Instructor Guidance

Instructor-guided training significantly increased the likelihood of checking the responsiveness of people experiencing cardiac arrest (RR 1.39, 95% CI 1.19-1.63; *P*<.001) and calling the emergency medical services (RR 1.11, 95% CI 1.00-1.24; *P*=.04). Although heterogeneity was high, instructor-guided training potentially increased mean compression depth. All of the instructor-guided intervention arms reported MDs favoring technology-based training (statistical significance in 4 out of 7 [57%] arms [30,34,38,42] and insignificance in 3 out of 7 [43%] arms [35,41,42]). The sole use of self-directed learning significantly decreased the likelihood of checking the airway (RR 0.84, 95% CI 0.75-0.94; *P*=.002) and correct hand position (RR 0.84, 95% CI 0.71-0.99; *P*=.04). Overall, instructor-guided training improved skills, whereas the sole use of self-directed learning yielded poorer skills.

#### Hands-on Practice

Despite high heterogeneity levels, hands-on practice potentially increased mean compression depth. Technology-based training interventions yielded significantly deeper chest compressions in 36% (4/11) of the intervention arms [30,34,38,42] and similar compression depths compared with standard training in 64% (7/11) of the intervention arms [27,31,35,40-42]. Similarly, although heterogeneity was high, hands-on practice potentially yielded slower compression rates than standard training; these reported compression rates were all within the guidelines of 100 to 120 compressions per minute. Training without hands-on practice significantly increased mean compression rate (MD 9.38, 95% CI 5.75-13.01; *P*<.001; *I*^2^=0%).

#### Training Modalities

**Figure 4:**
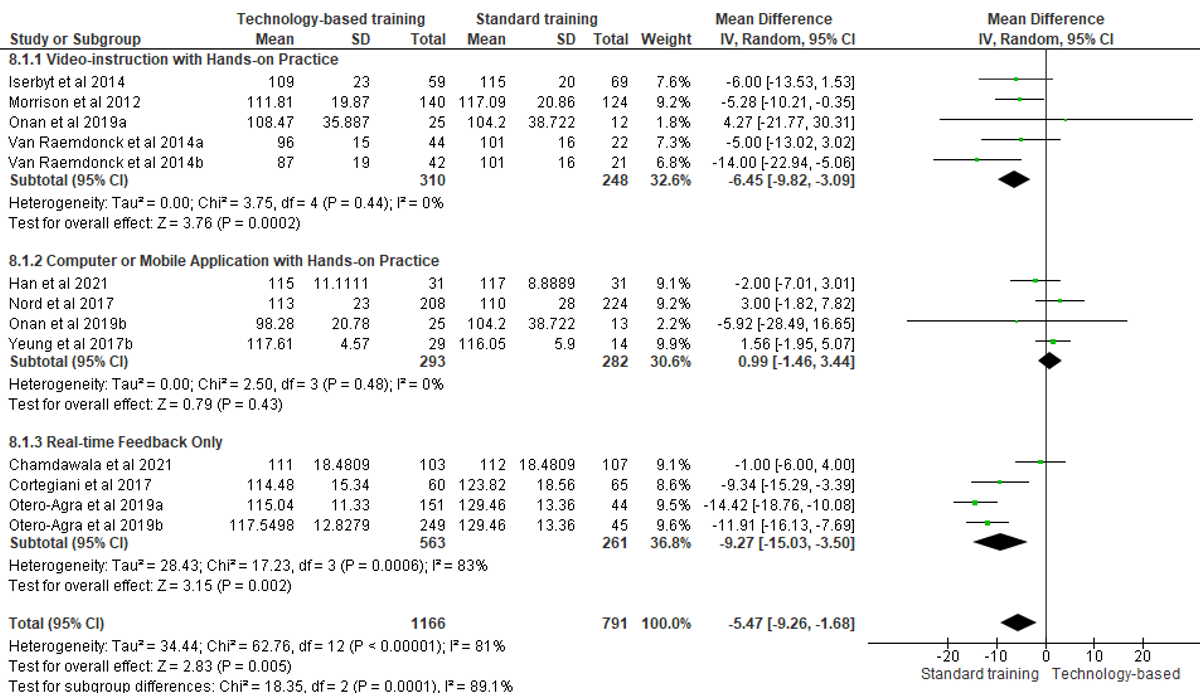
Subgroup analysis: mean compression rate after training.

Subgroup analyses of studies involving hands-on practice using different training modalities (Figure 4) revealed that video instruction with hands-on practice yielded significantly slower compression rates (MD –6.45, 95% CI –9.82 to –3.09; *P*<.001; *I*^2^=0%). Real-time feedback also potentially yielded slower compression rates, although heterogeneity was significantly high. Of the 4 arms involving real-time feedback that reported slower compression rates in the intervention groups, statistical significance was reached in 3 (75%) arms [32,34,35], whereas insignificance was reported in 1 (25%) arm [34].

Video instruction significantly decreased the likelihood of correct hand position (RR 0.78, 95% CI 0.61-1.00; *P*=.05; Table 5).

#### Retention

Technology-based training decreased the likelihood of correct compression depth (RR 0.76, 95% CI 0.59-0.97; *P*=.03; *I*^2^=0%; Table 3). Further analyses revealed that training with hands-on practice potentially significantly decreased mean compression rate compared with training without hands-on practice (*I*^2^=62.1%; *P*=.10; Table 5). Technology-based training also potentially decreased the likelihood of correct ventilation at 2 to 6 months (RR 0.64, 95% CI 0.41-0.99; *P*=.05; *I*^2^=78%; Table 3). Events of correct ventilation were significantly fewer in 60% (3/5) of the intervention arms [27,29] and similar to standard training in 40% (2/5) of the intervention arms [29,41].

### Effectiveness of Technology-Based Training on Knowledge

#### Posttraining Performance

Of the 17 RCTs, 6 (35%; arms: 8/24, 33%) involving 2253 students reported knowledge scores using questionnaires [26,29,33,34,41,42]. Owing to high heterogeneity (*I*^2^=89%; *P*<.001), subgroup analyses were performed (Multimedia Appendix 5). Instructor-guided training with hands-on practice potentially improved knowledge scores (Cohen *d*=0.45, 95% CI 0.13-0.78; *P*=.006; *I*^2^=84%). Only Chamdawala et al [34] reported insignificantly poorer knowledge scores after training with real-time feedback, and this contributed to the considerable heterogeneity. Of the 4 studies that reported MDs favoring technology-based training, statistical significance was achieved in 2 (50%) [29,41], whereas insignificance was reported in 1 (25%) [26]. *P* value was not reported by Onan et al [42].

Computer programs or mobile apps potentially improved knowledge scores (Cohen *d*=0.62, 95% CI 0.37-0.86; *P*<.001; *I*^2^=74%). The studies reported MDs favoring technology-based training. However, statistical significance was reported only in 33% (1/3) of the studies [41]. *P* values were not reported by Onan et al [42] and Reder et al [29].

Marchiori et al [39] lacked sufficient data to be included in the meta-analysis but reported that video game–based training significantly improved knowledge scores.

Overall, the effect of technology-based training on knowledge after training remains inconclusive. However, instructor guidance, hands-on practice, and computer programs or mobile apps potentially improved knowledge.

#### Retention

Meta-analysis on knowledge scores at 2 to 6 months pooled from 18% (3/17) of the trials (arms: 4/24, 17%), which involved 1862 students, revealed high heterogeneity (*I*^2^=89%; *P*<.001) [29,34,41]. Of these 3 studies, 1 (33%) [34] reported an insignificant difference in knowledge scores between training with real-time feedback and standard training, whereas 2 (67%) [29,41] reported significant improvements in knowledge scores. Overall, technology-based training potentially improved knowledge up to 6 months.

## Discussion

Our review showed that technology-based CPR training involving instructor guidance, hands-on practice, and real-time feedback yielded favorable outcomes for secondary school and high school students after the intervention. Technology-based training also potentially improved overall skills performance and knowledge at retention.

### CPR Skills

#### Posttraining Performance

Consistent with a recent meta-analysis conducted among laypeople and health care professionals [20], our study demonstrated that technology-based and standard CPR training produced comparable skills. The findings showed that instructor-guided training increased the likelihood of checking the responsiveness of people experiencing cardiac arrest and calling the emergency medical services and potentially increased compression depth. A meta-analysis [43] also reported better learning outcomes among health professionals who received instructor-supervised training compared with self-regulated learning. Instructors play important roles in increasing student motivation and providing personalized feedback on psychomotor skills [37,44]. These attributes, which are absent in self-directed learning, contribute to skill acquisition [45]. Consequently, the sole use of self-directed learning yielded poorer overall performance and reduced the likelihood of checking the airway and correct hand position. Similarly, a narrative review found that self-directed training potentially reduced overall CPR pass rates compared with instructor-led training [18]. Our findings suggest that instructor guidance remains essential for improved CPR performance in adolescents.

The findings revealed that technology-based training with hands-on practice potentially increased compression depth. In all of the included studies, the mean compression depth ranged from 30 mm to 53 mm, less than the maximum acceptable compression depth of 60 mm [46]. Adolescents often struggle with achieving adequate compression depths because of physical factors; for example, body weight [47]. Thus, practice is essential to acquire and reinforce proper compression techniques through trial and error. The incorporation of these participative and practical components boosted training success in adolescents [17]. In addition, video instruction with hands-on practice reduced mean compression rates, which were within the 2015 recommended guidelines [46] of 100 to 120 compressions per minute [31,40,42]. The study by Van Raemdonck et al [27] reported mean rates of <100 compressions per minute, considering that it applied the European Resuscitation Council 2005 guidelines, which accept 80 to 120 compressions per minute. Contrarily, training without hands-on practice increased compression rates. Without hands-on practice, instructions to *push hard, push fast* at *100 compressions per minute* may result in an overestimation of compression rates. Prior studies on technology-based training without practice also reported increased mean compression rates [48]. Overall, our findings suggest the importance of hands-on practice for improved CPR performance in adolescents.

In our review, real-time visual feedback improved overall compression quality, which comprises compression depth and rate, chest recoil, and hand position. Prior studies also reported that feedback devices contribute to improved chest compressions among health care professionals and adult laypeople [49]. Visual feedback allows trainees to contrast their performance against target parameters and correct themselves according to real-time performance data, improving skill acquisition. Real-time feedback also potentially yielded slower compression rates than standard training, and these mean rates were within 100 to 120 compressions per minute. The control groups in 67% (2/3) of the trials exceeded 120 compressions per minute [32,35]. Our findings suggest that real-time feedback improves chest compressions and possibly enhances adherence of compression rates to resuscitation guidelines.

However, video instruction reduced the likelihood of correct hand position. Similarly, an RCT [50] reported that video instruction training for health care staff yielded suboptimal hand position. As 67% (2/3) of the studies in our meta-analysis lacked clear descriptions of their instructional videos [27,42], it is challenging to examine explanations for this finding. One possible reason might be the inadequate emphasis on essential information; for example, anatomical landmarks for correct hand position in the instructional videos [31].

#### Retention

Technology-based training potentially improved overall performance at 6 months. Similarly, prior studies demonstrated improved skill retention in adolescents for up to 8 months after technology-based training [14]. However, technology-based training also reduced the likelihood of correct compression depth and potentially reduced the likelihood of correct ventilation at 2 to 6 months. Without refresher training, regression of skill performance from the second month is expected [16]. Skill regression in compression depth and ventilation may be more evident because these skills are considered challenging for adolescents [47]. Our findings suggest that regular refresher training is necessary to prevent skill decay.

#### Knowledge

Our findings were consistent with those of a past meta-analysis [20] that reported improved knowledge after digital resuscitation training among laypeople and health care professionals. In particular, instructor guidance, hands-on practice, and computer programs or mobile apps potentially yielded higher knowledge scores. Instructors improve students’ theoretical understanding by simplifying complex concepts, identifying individual areas of weaknesses, and promptly clarifying doubts [51]. Hands-on practice allows students to put theory into practice and enhance knowledge acquisition and retention [17]. The participative features in computer programs or mobile apps increase students’ interest and help them to grasp concepts quickly [52]. Students can access training materials via electronic devices easily to reinforce knowledge and improve knowledge retention [14].

However, knowledge questionnaires were not standardized across the studies. Recently, a questionnaire assessing adolescents’ CPR knowledge was developed and validated [53]. Adoption of standardized assessment by future studies will be beneficial because intervention effects on knowledge can be easily compared across studies. In addition, learning theories improved CPR knowledge [20]. However, only Iserbyt et al [31] in this review used learning theory to guide their intervention.

### Strengths and Limitations

The strengths of this review include an extensive search in multiple bibliographic databases and comprehensive synthesis of results. However, the review was limited by the low quality of the included studies. Most (16/17, 94%) of the studies inadequately reported or took measures to reduce selection and performance biases. In addition, variations in intervention designs across the studies increased heterogeneity; for instance, videos and computer programs or mobile apps may emphasize theoretical knowledge, whereas interventions involving real-time feedback focused on CPR skills. Furthermore, several (11/17, 65%) of the technology-based interventions involved active interaction and engagement with students, whereas others (7/17, 41%) involved passive learning through videos. These variations made it challenging to draw conclusions on training elements required for optimal effectiveness. Finally, this review only included trials published in English.

### Implications for Research

More high-quality RCTs with clear descriptions of study procedures—for example, allocation concealment and blinding of participants and personnel—are needed. These efforts will improve the credibility of evidence and contribute to stronger conclusions on the effectiveness of technology-based training for adolescents. Future studies should consider incorporating learning theories to guide their interventions [20]. Technology-based training can be considered a routine refresher training modality in schools for future research.

### Implications for Practice

Overall, technology-based training demonstrated equivalence or improvements in skills and knowledge after training and at retention when compared with standard training among adolescents. From an educational perspective, the noninferiority of technology-based training offers a desirable alternative to standard training. Schools can consider using videos, computer programs, or mobile apps for self-directed theoretical instruction. However, instructor guidance and hands-on practice are still necessary components of training. Real-time feedback devices may also be used to increase students’ compliance to resuscitation guidelines. Such a blended learning approach, comprising technology-based resources and face-to-face teaching, may reduce class time and reliance on instructor availability and increase schools’ capacity for wider training dissemination. Furthermore, refresher training should focus on challenging skills; for example, compression depth and ventilation.

### Conclusions

This review explored the use of technology-based training as an alternative to standard CPR training among secondary school and high school students. Our findings supported the use of technology-based components such as videos, computer programs, or mobile apps for self-directed theoretical instruction; these components potentially improve skills and knowledge retention. However, instructor guidance, hands-on practice, and real-time feedback are still necessary components of training to achieve better learning outcomes for adolescents. Such a blended learning approach may reduce class time and reliance on instructor availability. Regular refresher training is necessary for challenging skills such as compression depth and ventilation. Caution must be exercised when interpreting the results of this review because of the high heterogeneity of intervention characteristics. The overall low quality of evidence indicated the need for high-quality RCTs with large sample sizes and follow-up data.

## Appendix

Multimedia Appendix 1 Database search terms and keywords.

Multimedia Appendix 2 Descriptions of interventions.

Multimedia Appendix 3 Grading of Recommendations Assessment, Development, and Evaluation.

Multimedia Appendix 4 Results of subgroup analyses for skills and knowledge.

Multimedia Appendix 5 Subgroup analyses of knowledge after training.

## Abbreviations

CPR: cardiopulmonary resuscitation
MD: mean difference
OCHA: out-of-hospital cardiac arrest
PRISMA: Preferred Reporting Items for Systematic Reviews and Meta-Analyses
RCT: randomized controlled trial
RR: risk ratio
