# Supplementary material for: The Effectiveness of Technology-Based Cardiopulmonary Resuscitation Training on the Skills and Knowledge of Adolescents: Systematic Review and Meta-analysis
Source: J Med Internet Res. 2022 Dec 15;24(12):e36423. doi: 10.2196/36423 (PMC9801268; doi:10.2196/36423)
Supplement: Multimedia Appendix 1 [file jmir_v24i12e36423_app1.pdf]

Database	Index & Keyword Terms
PubMed	#1 Schoolchildren[Title/Abstract] OR "school children"[Title/Abstract] OR student*[Title/Abstract] OR child*[Title/Abstract] OR adolescent*[Title/Abstract] OR "middle school*"[Title/Abstract] OR "high school*"[Title/Abstract] OR "secondary school*"[Title/Abstract]<br>#2 "Adolescent"[Mesh] OR "Schools"[Mesh:NoExp] OR "Students"[Mesh:NoExp] OR "Child"[Mesh:NoExp]<br>#3 #1 OR #2<br>#4 "cardiopulmonary resuscitation"[Title/Abstract] OR CPR[Title/Abstract] OR "basic cardiac life support"[Title/Abstract] OR BCLS[Title/Abstract] OR "basic life support"[Title/Abstract] OR BLS[Title/Abstract] OR "basic life"[Title/Abstract] OR resuscit*[Title/Abstract] OR "automated external defibrillator*"[Title/Abstract] OR AED[Title/Abstract]<br>#5 "Cardiopulmonary Resuscitation"[Mesh:NoExp] OR "Defibrillators"[Mesh:NoExp]<br>#6 #4 OR #5<br>#7 Train*[Title/Abstract] OR teach*[Title/Abstract] OR educat*[Title/Abstract]<br>#8 "Education"[Mesh:NoExp] OR "Teaching"[Mesh]<br>#9 #7 OR #8<br>#10 #3 AND #6 AND #9
EMBASE	((schoolchildren:ti,ab OR 'school children':ti,ab OR student*:ti,ab OR child*:ti,ab OR adolescent*:ti,ab OR 'middle school*':ti,ab OR 'high school*':ti,ab OR 'secondary school*':ti,ab) OR ('school child'/exp OR 'adolescent'/de OR 'high school'/exp OR 'middle school'/exp OR 'school'/exp OR 'high school student'/exp OR 'middle school student'/exp)) AND (('cardiopulmonary resuscitation':ti,ab OR cpr:ti,ab OR 'basic cardiac life support':ti,ab OR bcls:ti,ab OR 'basic life support':ti,ab OR bls:ti,ab OR 'basic life':ti,ab OR resuscit*:ti,ab OR 'automated external defibrillator*':ti,ab OR aed:ti,ab) OR ('resuscitation'/exp OR 'automated external defibrillator'/exp)) AND ((train*:ti,ab OR teach*:ti,ab OR educat*:ti,ab) OR ('training'/exp OR 'teaching'/exp OR 'education'/exp))

Database	Index & Keyword Terms
Cochrane Library	#1 MeSH descriptor: [Adolescent] explode all trees<br>#2 MeSH descriptor: [Schools] this term only<br>#3 MeSH descriptor: [Students] this term only<br>#4 MeSH descriptor: [Child] this term only<br>#5 #1 OR #2 OR #3 OR #4<br>#6 (Schoolchildren OR "school children" OR student* OR child* OR adolescent* OR middle school* OR high school* OR secondary school*):ti,ab,kw<br>#7 #5 OR #6<br>#8 MeSH descriptor: [Cardiopulmonary Resuscitation] this term only<br>#9 MeSH descriptor: [Defibrillators] this term only<br>#10 #8 OR #9<br>#11 ("cardiopulmonary resuscitation" OR CPR OR "basic cardiac life support" OR BCLS OR "basic life support" OR BLS OR "basic life" OR resuscit* OR automated external defibrillator* OR AED):ti,ab,kw<br>#12 #10 OR #11<br>#13 MeSH descriptor: [Teaching] this term only<br>#14 MeSH descriptor: [Education] this term only<br>#15 (Train* OR teach* OR educat*):ti,ab,kw<br>#16 #13 OR #14 OR #15<br>#17 #7 AND #12 AND #16
Ovid MEDLINE	#1 (Schoolchildren or school children or student* or child* or adolescent* or middle school* or high school* or secondary school*).ab,ti.<br>#2 exp Adolescent/ or Schools/ or Students/ or Child/<br>#3 1 or 2<br>#4 (cardiopulmonary resuscitation or CPR or basic cardiac life support or BCLS or basic life support or BLS or basic life or resuscit* or automated external defibrillator* or AED).ab,ti.<br>#5 Cardiopulmonary Resuscitation/ or Defibrillators/<br>#6 4 or 5<br>#7 (Train* or teach* or educat*).ab,ti.<br>#8 education/ or exp teaching/<br>#9 7 or 8<br>#10 3 and 6 and 9

Database	Index & Keyword Terms
CINAHL	S1 TI (Schoolchildren OR “school children” OR student* OR child* OR adolescent* OR “middle school*” OR “high school*” OR “secondary school*”) OR AB (Schoolchildren OR “school children” OR student* OR child* OR adolescent* OR “middle school*” OR “high school*” OR “secondary school*”)<br>S2 MH (MH "Adolescence") OR (MH "Students, Middle School") OR (MH "Students, High School") OR (MH "Schools, Middle") OR (MH "Schools, Secondary")<br>S3 S1 OR S2<br>S4 TI (“cardiopulmonary resuscitation” OR CPR OR “basic cardiac life support” OR BCLS OR “basic life support” OR BLS OR “basic life” OR resuscit* OR “automated external defibrillator*” OR AED) OR AB (“cardiopulmonary resuscitation” OR CPR OR “basic cardiac life support” OR BCLS OR “basic life support” OR BLS OR “basic life” OR resuscit* OR “automated external defibrillator*” OR AED)<br>S5 MH (MH "Resuscitation, Cardiopulmonary") OR (MH "Bystander CPR") OR (MH "Defibrillators, Automated External")<br>S6 S4 OR S5<br>S7 TI (Train* OR teach* OR educat*) OR AB (Train* OR teach* OR educat*)<br>S8 MH (MH "Teaching+") OR (MH "Teaching Methods+") OR (MH "Education")<br>S9 S7 OR S8<br>S10 S3 AND S6 AND S9
PsycINFO	#1 (Schoolchildren or 'school children' or student* or child* or adolescent* or 'middle school*' or 'high school*' or 'secondary school*').ab,ti.<br>#2 high schools/ or junior high schools/ or middle schools/ or high school students/ or junior high school students/ or middle school students/<br>#3 1 or 2<br>#4 ('cardiopulmonary resuscitation' or CPR or 'basic cardiac life support' or BCLS or 'basic life support' or BLS or 'basic life' or resuscit* or 'automated external defibrillator*' or AED).ab,ti.<br>#5 cpr/<br>#6 4 or 5<br>#7 (Train* or teach* or educat*).ab,ti.<br>#8 training/ or teaching/ or education/ or high school education/ or secondary education/ or middle school education/<br>#9 7 or 8<br>#10 3 and 6 and 9

Database	Index & Keyword Terms
ERIC	((ab(Schoolchildren OR "school children" OR student* OR child* OR adolescent* OR "middle school*" OR "high school*" OR "secondary school*") OR ti(Schoolchildren OR "school children" OR student* OR child* OR adolescent* OR "middle school*" OR "high school*" OR "secondary school*")) OR (MAINSUBJECT.EXACT("Late Adolescents") OR MAINSUBJECT.EXACT("Secondary Education") OR MAINSUBJECT.EXACT("Middle Schools") OR MAINSUBJECT.EXACT("Early Adolescents") OR MAINSUBJECT.EXACT("High Schools") OR MAINSUBJECT.EXACT("Preadolescents") OR MAINSUBJECT.EXACT("Middle School Students") OR MAINSUBJECT.EXACT("High School Students") OR MAINSUBJECT.EXACT("Adolescents") OR MAINSUBJECT.EXACT("Intermediate Grades"))) AND ((ab("cardiopulmonary resuscitation" OR CPR OR "basic cardiac life support" OR BCLS OR "basic life support" OR BLS OR "basic life" OR resuscit* OR "automated external defibrillator*" OR AED) OR ti("cardiopulmonary resuscitation" OR CPR OR "basic cardiac life support" OR BCLS OR "basic life support" OR BLS OR "basic life" OR resuscit* OR "automated external defibrillator*" OR AED)) OR MAINSUBJECT.EXACT("First Aid")) AND (ab(Train* OR teach* OR educat*) OR ti(Train* OR teach* OR educat*))
ProQuest Dissertations and Theses	((ab(Schoolchildren OR "school children" OR student* OR child* OR adolescent* OR "middle school*" OR "high school*" OR "secondary school*") OR ti(Schoolchildren OR "school children" OR student* OR child* OR adolescent* OR "middle school*" OR "high school*" OR "secondary school*")) OR (MAINSUBJECT.EXACT("Middle schools") OR MAINSUBJECT.EXACT("Teenagers") OR MAINSUBJECT.EXACT("Middle school students") OR MAINSUBJECT.EXACT("Secondary school students") OR MAINSUBJECT.EXACT("Secondary schools"))) AND ((ab("cardiopulmonary resuscitation" OR CPR OR "basic cardiac life support" OR BCLS OR "basic life support" OR BLS OR "basic life" OR resuscit* OR "automated external defibrillator*" OR AED) OR ti("cardiopulmonary resuscitation" OR CPR OR "basic cardiac life support" OR BCLS OR "basic life support" OR BLS OR "basic life" OR resuscit* OR "automated external defibrillator*" OR AED)) OR (MAINSUBJECT.EXACT("Cardiopulmonary resuscitation CPR") OR MAINSUBJECT.EXACT("Lifesaving") OR MAINSUBJECT.EXACT("First aid"))) AND (ab(Train* OR teach* OR educat*) OR ti(Train* OR teach* OR educat*))

Database	Index & Keyword Terms
Scopus	(TITLE-ABS-KEY (schoolchildren OR "school children" OR student* OR child* OR adolescent* OR "middle school*" OR "high school*" OR "secondary school*")) AND (TITLE-ABS-KEY ("cardiopulmonary resuscitation" OR cpr OR "basic cardiac life support" OR bcls OR "basic life support" OR bls OR "basic life" OR resuscit* OR "automated external defibrillator*" OR aed)) AND (TITLE-ABS-KEY (train* OR teach* OR educat*))
