# Supplementary material for: The Effectiveness of Technology-Based Cardiopulmonary Resuscitation Training on the Skills and Knowledge of Adolescents: Systematic Review and Meta-analysis
Source: J Med Internet Res. 2022 Dec 15;24(12):e36423. doi: 10.2196/36423 (PMC9801268; doi:10.2196/36423)
Supplement: Multimedia Appendix 2 [file jmir_v24i12e36423_app2.pdf]

Author (Year)	Intervention	Ventilation	AED	Hands-on Practice	Individual/ Shared Manikin	Instructor	Duration	No. of Sessions	Resuscitation Guidelines
Beskind et al.[28]	Brief video produced by the University of Arizona Sarver Heart Center<br>Video describes how to perform CCO CPR	No	Yes	No	NA	NIL	1.5 mins	1	AHA & ERC 2010
Chamdawala et al.[34]	Practical training with QCPR real-time visual feedback manikin	No	No	Yes	Unclear	Research assistant	2 mins	1	AHA 2015
Cortegiani et al.[35]	Interactive frontal lesson, instructor demonstration, and QCPR real-time feedback	No	Yes	Yes	Individual	Certified instructor	30 mins	1	ERC 2015
Cuijpers et al.[36]	Online internet-based learning with instructor-led training, followed by another instructor-led training 2 weeks later	Yes	NR	Yes	Individual	NR	1st session: 1-2 hrs<br>2nd session: 2 hrs	2	ERC 2010
Doucet et al.[37]	Information sheet, app on tablet PCs, video, and peer feedback<br>App involves self-instructional video, simulation, and evaluation checklist	Yes	Yes	Yes	Shared (Manikin to student ratio unclear)	NIL	40 mins	1	ERC 2015
Han et al.[38]	E-learning on Kahoot! with assessments, followed by videoconference for hands-on practice	No	Yes	Yes	Individual	CPR instructor	60 mins	1	AHA 2020
Iserbyt et al.[31]	Videos on tablet PCs, doer-helper relationship, spatial contiguity principle<br>Based on multimedia learning theory	Yes	No	Yes	Shared (1 manikin to 2 students)	PE teacher (facilitate only)	5 mins introduction<br>20 mins learning<br>10 mins peer assessment	1	ERC 2010

Abbreviations: AED, Automated External Defibrillator; AHA, American Heart Association; App, Application; CCO, Chest-Compressions-Only; CPR, Cardiopulmonary Resuscitation; ERC, European Resuscitation Council; ILCOR, International Liaison Committee of Resuscitation; NA, Not Applicable; NIL, None; NR, Not Reported; PCs, Personal Computers; QCPR, Quality CPR.

Author (Year)	Description of Intervention	Ventilation	AED	Hands-on Practice	Individual/ Shared Manikin	Instructor	Duration	No. of Sessions	Resuscitation Guidelines
Marchiori et al.[39]	Computer video game	No	Yes	No	NA	NIL	45 mins	1	ILCOR 2010
Morrison et al.[40]	Video-instruction with instructional booklet (CPR) and instructor-led (AED)<br>CPR self-instruction kit costs $35/kit	Yes	Yes	Yes	Individual	Instructor for AED only	22 mins video<br>Total training 45 mins	1	Heart & Stroke Foundation of Canada
Nord et al.[41]	Web course followed by instructor-led training 7 days later.<br>Web course involves videos, animations, quizzes, storytelling.	Yes	No	Yes	Individual	School teachers (CPR trained)	20-30 mins web course<br>30-50 mins instructor-led	2	ERC 2010
Norman[26]	Videocassette of full and precise instructor demonstration.	Yes	No	Yes	Unclear	CPR instructor	27 mins	1	AHA
Onan et al.[42]	A: Video-instruction with instructor demonstration;<br>B: Including mobile-assisted feedback (PocketCPR app).<br>Video instruction & demonstration, with narration by course teacher.	Yes	No	Yes	Shared (1 manikin to 5 students)	Course teacher	7 mins video<br>13 mins teaching<br>60 mins practice	1	AHA, Emergency cardiovascular care guidelines, Ministry of National Education
Otero-Agra et al.[32]	A: Group training with QCPR and competition;<br>B: Individual training using QCPR, with or without grading.	No	No	Yes	Individual	CPR Instructor	50 mins	1	ERC 2015
Reder et al.[29]	A: Interactive computer session;<br>B: Interactive computer session, followed by instructor-led practice.<br>Computer software (vignettes & games) produced by National Center for Early Defibrillation.	Yes	Yes	A: No<br>B: Yes	A: NA<br>B: Shared (1 manikin to 7 students)	A: NIL<br>B: Medical students	A: 45 mins<br>B: 45 mins computer session, 45 mins instructor-led practice	A: 1<br>B: 2	AHA

Abbreviations: AED, Automated External Defibrillator; AHA, American Heart Association; App, Application; CPR, Cardiopulmonary Resuscitation; ERC, European Resuscitation Council; NA, Not Applicable; NIL, None; NR, Not Reported; QCPR, Quality CPR.

Author (Year)	Description of Intervention	Ventilation	AED	Hands-on Practice	Individual/ Shared Manikin	Instructor	Duration	No. of Sessions	Resuscitation Guidelines
Rezaei et al.[33]	Recorded film teaching.	Yes	No	No	NA	Teacher	NR	1	Unclear
Van Raemdonck et al.[27]	A: Video-instruction and practice on standard manikin, peer feedback;<br>B: Video-instruction and practice on low-cost materials (foam dice & plastic bag), peer feedback.<br>Video instruction & demonstration. 8 mins practice session with metronome and task cards.	Yes	No	Yes	Shared (1 manikin to 4 students)	NIL	50 mins	1	ERC 2015
Yeung et al.[30]	A: Lifesaver app on handheld tablet (interactive game);<br>B: Lifesaver app followed by instructor-led training.<br>Game-in-film format, real-life scenarios, simulation.	Yes	Yes	A: No<br>B: Yes	A: NA<br>B: Shared (1 manikin to 6 students)	A: No<br>B: CPR instructor	NR	A: 1<br>B: 2	ERC 2015

Abbreviations: AED, Automated External Defibrillator; App, Application; CPR, Cardiopulmonary Resuscitation; ERC, European Resuscitation Council; NA, Not Applicable; NIL, None; NR, Not Reported
