# Supplementary material for: The Effectiveness of Technology-Based Cardiopulmonary Resuscitation Training on the Skills and Knowledge of Adolescents: Systematic Review and Meta-analysis
Source: J Med Internet Res. 2022 Dec 15;24(12):e36423. doi: 10.2196/36423 (PMC9801268; doi:10.2196/36423)
Supplement: Multimedia Appendix 3 [file jmir_v24i12e36423_app3.pdf]

Certainty assessment							№ of patients		Effect		Certainty	Importance
**№ of studies**	**Study design**	**Risk of bias**	**Inconsistency**	**Indirectness**	**Imprecision**	**Other considerations**	**Technology-based training**	**Standard training**	**Relative (95% CI)**	**Absolute (95% CI)**		
**Overall Skill Performance (Post-training)**												
6	randomised trials	serious [a,b]	serious [c]	serious [d]	serious [c,e,f]	none	602	519	-	SMD **0.01 SD higher** (0.4 lower to 0.42 higher)	⊕◯◯◯ VERY LOW	IMPORTANT
**Knowledge Scores (Post-training)**												
6	randomised trials	serious [a,g]	serious [c]	serious [d]	serious [c,h]	none	1254	999	-	SMD **0.6 SD higher** (0.28 higher to 0.93 higher)	⊕◯◯◯ VERY LOW	IMPORTANT

CI: Confidence interval; SMD: Standardised mean difference

Explanations:
a. All studies did not report allocation concealment
b. 3 studies did not blind or adequately report blinding of outcome assessors
c. Significant heterogeneity
d. Technology-based training varied across studies
e. 3 studies had small sample sizes
f. Wide confidence interval
g. 1 study did not report pre-determined outcomes, data obtained from author
h. 3 studies had small sample sizes
