# Supplementary material for: The Effectiveness of Technology-Based Cardiopulmonary Resuscitation Training on the Skills and Knowledge of Adolescents: Systematic Review and Meta-analysis
Source: J Med Internet Res. 2022 Dec 15;24(12):e36423. doi: 10.2196/36423 (PMC9801268; doi:10.2196/36423)
Supplement: Multimedia Appendix 4 [file jmir_v24i12e36423_app4.pdf]

| Subgroup analyses | | Number of comparisons | Effect estimate | 95%CI | Subgroup effect Z | Subgroup effect p-value | $I^2$ | Subgroup differences $I^2$ | Subgroup differences p-value |
|---|---|---|---|---|---|---|---|---|---|
| **Overall Performance Scores (Post-training)**: 6 trials (8 arms) | | | | | | | | | |
| Presence of hand-on practice | Hands-on practice | 7 | 0.13 | -0.29–0.55 | 0.60 | 0.55 | 89 | 87.5 | 0.005*[a] |
| | Without practical training | 1 | -1.07 | -1.79–-0.35 | 2.92 | 0.003*[a] | NA | | |
| Training modality | Video-instruction | 2 | -0.08 | -0.45–0.29 | 0.44 | 0.66 | 31 | 0 | 0.74 |
| | Computer/mobile | 6 | 0.04 | -0.55–0.62 | 0.12 | 0.90 | 92 | | |
| | Real-time feedback only | 0 | - | - | - | - | - | | |
| Presence of instructor | Self-directed learning | 2 | -0.74 | -1.02–-0.45 | 5.02 | <0.00001* | 0 | 92.8% | 0.0002* |
| | Instructor-guided | 6 | 0.28 | -0.17–0.73 | 1.22 | 0.22 | 88 | | |
| **Checking Responsiveness (Post-training)**: 5 trials (6 arms) | | | | | | | | | |
| Presence of hand-on practice | Hands-on practice | 5 | 1.08 | 0.83–1.40 | 0.58 | 0.56 | 87 | 74.2 | 0.05*[a] |
| | Without practical training | 1 | 1.61 | 1.20–2.16 | 3.14 | 0.002*[a] | NA | | |
| Training modality | Video-instruction | 3 | 1.12 | 0.71–1.78 | 0.49 | 0.62 | 84 | 0 | 0.82 |
| | Computer/mobile | 3 | 1.21 | 0.78–1.87 | 0.84 | 0.40 | 93 | | |
| | Real-time feedback only | 0 | - | - | - | - | - | | |
| Presence of instructor | Self-directed learning | 3 | 1.07 | 0.83–1.38 | 0.50 | 0.61 | 86 | 67 | 0.08* |
| | Instructor-guided | 3 | 1.39 | 1.19–1.63 | 4.10 | <0.0001* | 0 | | |
| **Checking Airway (Post-training):** 5 trials (6 arms) | | | | | | | | | |
| Presence of hand-on practice | Hands-on practice | 4 | 0.94 | 0.74–1.19 | 0.55 | 0.59 | 69 | 0 | 0.94 |
| | Without practical training | 2 | 0.92 | 0.66–1.28 | 0.50 | 0.62 | 56 | | |
| Training modality | Video-instruction | 2 | 1.07 | 0.77–1.48 | 0.39 | 0.70 | 0 | 0 | 0.39 |
| | Computer/mobile | 4 | 0.90 | 0.74–1.11 | 0.99 | 0.32 | 73 | | |
| | Real-time feedback only | 0 | - | - | - | - | - | | |
| Presence of instructor | Self-directed learning | 4 | 0.84 | 0.75–0.94 | 3.05 | 0.002* | 0 | 4.6 | 0.31 |
| | Instructor-guided | 2 | 1.02 | 0.71–1.48 | 0.13 | 0.90 | 63 | | |
| **Calling EMS/ Help (Post-training):** 6 trials (7 arms) | | | | | | | | | |
| Presence of hand-on practice | Hands-on practice | 5 | 1.02 | 0.88–1.18 | 0.29 | 0.77 | 66 | 4.6 | 0.31 |
| | Without practical training | 2 | 1.41 | 0.77–2.58 | 1.12 | 0.26 | 82 | | |
| Training modality | Video-instruction | 4 | 1.18 | 0.86–1.63 | 1.03 | 0.30 | 63 | 0 | 0.56 |
| | Computer/mobile | 3 | 1.05 | 0.85–1.31 | 0.47 | 0.64 | 88 | | |
| | Real-time feedback only | 0 | - | - | - | - | - | | |
| Presence of instructor | Self-directed learning | 4 | 1.10 | 0.85–1.43 | 0.72 | 0.47 | 85 | 0 | 0.93 |
| | Instructor-guided | 3 | 1.11 | 1.00–1.24 | 2.01 | 0.04* | 0 | | |

a: significance for 1 trial only

| Subgroup analyses | | Number of comparisons | Effect estimate | 95%CI | Subgroup effect | | $I^2$ | Subgroup differences | |
|---|---|---|---|---|---|---|---|---|---|
| | | | | | Z | p-value | | $I^2$ | p-value |
| **Mean Compression Depth (Post-training):** 10 trials (13 arms) | | | | | | | | | |
| Presence of hand-on practice | Hands-on practice | 11 | 2.20 | 0.08–4.32 | 2.03 | 0.04* | 79 | 70.6 | 0.07* |
| | Without practical training | 2 | -6.52 | –15.53–2.50 | 1.42 | 0.16 | 94 | | |
| Training modality | Video-instruction | 6 | -0.80 | -2.41–0.82 | 0.97 | 0.33 | 0 | 47.1 | 0.15 |
| | Computer/mobile | 5 | 2.34 | -5.36–10.04 | 0.59 | 0.55 | 98 | | |
| | Real-time feedback only | 2 | 3.01 | -0.71–6.73 | 1.59 | 0.11 | 72 | | |
| Presence of instructor | Self-directed learning | 6 | -3.16 | -8.17–1.85 | 1.23 | 0.22 | 93 | 83.9 | 0.01* |
| | Instructor-guided | 7 | 3.94 | 1.50–6.37 | 3.17 | 0.002* | 78 | | |
| **Mean Compression Depth (Retention):** 6 trials (8 arms) | | | | | | | | | |
| Presence of hand-on practice | Hands-on practice | 6 | 1.48 | -3.30–6.26 | 0.61 | 0.54 | 95 | 0 | 0.37 |
| | Without practical training | 2 | -1.14 | -4.37–2.09 | 0.69 | 0.49 | 53 | | |
| Training modality | Video-instruction | 4 | -1.55 | -3.50–0.40 | 1.56 | 0.12 | 0 | 86.9 | 0.0005*[a] |
| | Computer/mobile | 3 | 2.33 | -5.27–9.93 | 0.60 | 0.55 | 98 | | |
| | Real-time feedback only | 1 | 4.00 | 2.00–6.00 | 3.92 | <0.0001* | NA | | |
| Presence of instructor | Self-directed learning | 5 | -0.75 | -2.13–0.63 | 1.07 | 0.29 | 0 | 30.9 | 0.23 |
| | Instructor-guided | 3 | 3.64 | -3.38–10.66 | 1.02 | 0.31 | 98 | | |
| **Correct Compression Depth (Post-training):** 6 trials (8 arms) | | | | | | | | | |
| Presence of hand-on practice | Hands-on practice | 8 | 1.04 | 0.90–1.21 | 0.54 | 0.59 | 43 | NA | NA |
| | Without practical training | 0 | - | - | - | - | - | | |
| Training modality | Video-instruction | 3 | 0.92 | 0.59–1.43 | 0.36 | 0.72 | 0 | 80.6 | 0.006*[a] |
| | Computer/mobile | 3 | 0.90 | 0.77–1.05 | 1.35 | 0.18 | 0 | | |
| | Real-time feedback only | 2 | 1.25 | 1.09–1.43 | 3.23 | 0.001*[a] | NA | | |
| Presence of instructor | Self-directed learning | 4 | 0.91 | 0.76–1.08 | 1.05 | 0.29 | 0 | 54.2 | 0.14 |
| | Instructor-guided | 4 | 1.11 | 0.91–1.34 | 1.04 | 0.30 | 54 | | |
| **Mean Compression Rate (Post-training):** 11 trials (15 arms) | | | | | | | | | |
| Presence of hand-on practice | Hands-on practice | 13 | -5.47 | -9.26–-1.68 | 2.83 | 0.005* | 81 | 96.7 | <0.00001* |
| | Without practical training | 2 | 9.38 | 5.75–13.01 | 5.07 | <0.00001* | 0 | | |
| Training modality | Video-instruction | 6 | -3.79 | -9.89–2.32 | 1.22 | 0.22 | 66 | 83.3 | 0.002* |
| | Computer/mobile | 5 | 2.75 | -1.65–7.14 | 1.22 | 0.22 | 72 | | |
| | Real-time feedback only | 4 | -9.27 | -15.03–-3.50 | 3.15 | 0.002* | 83 | | |
| Presence of instructor | Self-directed learning | 6 | -1.59 | -9.42–6.25 | 0.40 | 0.69 | 88 | 0 | 0.53 |
| | Instructor-guided | 9 | -4.61 | -9.71–0.49 | 1.77 | 0.08 | 86 | | |

a: significance for 1 trial only

| Subgroup analyses | | Number of comparisons | Effect estimate | 95%CI | Subgroup effect Z | Subgroup effect p-value | $I^2$ | Subgroup differences $I^2$ | Subgroup differences p-value |
|---|---|---|---|---|---|---|---|---|---|
| **Mean Compression Rate (Retention):** 6 trials (8 arms) | | | | | | | | | |
| Presence of hand-on practice | Hands-on practice | 6 | -3.88 | -9.79–2.03 | 1.29 | 0.20 | 86 | 62.1 | 0.10* |
| | Without practical training | 2 | 1.80 | –1.67–5.27 | 1.02 | 0.31 | 0 | | |
| Training modality | Video-instruction | 4 | -1.96 | -6.04–2.13 | 0.94 | 0.35 | 0 | 0 | 0.96 |
| | Computer/mobile | 3 | -3.55 | -14.36–7.26 | 0.64 | 0.52 | 95 | | |
| | Real-time feedback only | 1 | -2.00 | -6.00–2.00 | 0.98 | 0.33 | NA | | |
| Presence of instructor | Self-directed learning | 5 | 0.08 | -2.67–2.83 | 0.06 | 0.95 | 0 | 0 | 0.33 |
| | Instructor-guided | 3 | -4.84 | -14.33–4.64 | 1.00 | 0.32 | 94 | | |
| **Correct Compression Rate (Post-training):** 5 trials (7 arms) | | | | | | | | | |
| Presence of hand-on practice | Hands-on practice | 7 | 0.89 | 0.75–1.07 | 1.22 | 0.22 | 38 | NA | NA |
| | Without practical training | 0 | - | - | - | - | - | | |
| Training modality | Video-instruction | 4 | 0.91 | 0.76–1.08 | 1.09 | 0.28 | 0 | 0 | 0.83 |
| | Computer/mobile | 3 | 0.95 | 0.61–1.50 | 0.20 | 0.84 | 72 | | |
| | Real-time feedback only | 0 | - | - | - | - | - | | |
| Presence of instructor | Self-directed learning | 4 | 0.91 | 0.80–1.04 | 1.41 | 0.16 | 0 | 0 | 0.53 |
| | Instructor-guided | 3 | 1.27 | 0.45–3.60 | 0.45 | 0.65 | 74 | | |
| **Correct Hand Position (Post-training):** 7 trials (10 arms) | | | | | | | | | |
| Presence of hand-on practice | Hands-on practice | 9 | 0.96 | 0.85–1.07 | 0.75 | 0.45 | 41 | 66.4 | 0.08*[a] |
| | Without practical training | 1 | 0.79 | 0.66–0.95 | 2.49 | 0.01*[a] | NA | | |
| Training modality | Video-instruction | 4 | 0.78 | 0.61–1.00 | 1.92 | 0.05* | 0 | 9.3 | 0.33 |
| | Computer/mobile | 5 | 0.99 | 0.82–1.18 | 0.16 | 0.87 | 66 | | |
| | Real-time feedback only | 1 | 0.93 | 0.84–1.02 | 1.53 | 0.13 | NA | | |
| Presence of instructor | Self-directed learning | 5 | 0.84 | 0.71–0.99 | 2.10 | 0.04* | 48 | 64.1 | 0.09* |
| | Instructor-guided | 5 | 1.11 | 0.83–1.47 | 0.71 | 0.48 | 74 | | |
| **Correct Ventilation (Post-training):** 8 trials (11 arms) | | | | | | | | | |
| Presence of hand-on practice | Hands-on practice | 9 | 0.89 | 0.77–1.03 | 1.52 | 0.13 | 9 | 0 | 0.91 |
| | Without practical training | 2 | 0.96 | 0.30–3.04 | 0.07 | 0.94 | 95 | | |
| Training modality | Video-instruction | 5 | 0.84 | 0.43–1.65 | 0.50 | 0.62 | 71 | 0 | 0.96 |
| | Computer/mobile | 6 | 0.83 | 0.64–1.08 | 1.40 | 0.16 | 70 | | |
| | Real-time feedback only | 0 | - | - | - | - | - | | |
| Presence of instructor | Self-directed learning | 6 | 0.79 | 0.53–1.16 | 1.20 | 0.23 | 81 | 0 | 0.35 |
| | Instructor-guided | 5 | 0.97 | 0.81–1.16 | 0.37 | 0.71 | 0 | | |

a: significance for 1 trial only

| Subgroup analyses | | Number of comparisons | Effect estimate | 95%CI | Subgroup effect | | $I^2$ | Subgroup differences | |
|---|---|---|---|---|---|---|---|---|---|
| | | | | | Z | p-value | | $I^2$ | p-value |
| **Knowledge (Post-training):** 6 trials (8 arms) | | | | | | | | | |
| Presence of hand-on practice | Hands-on practice | 6 | 0.45 | 0.13–0.78 | 2.73 | 0.006* | 84 | 0 | 0.43 |
| | Without practical training | 2 | 1.12 | -0.49–2.73 | 1.37 | 0.17 | 97 | | |
| Training modality | Video-instruction | 3 | 0.80 | -0.39–1.99 | 1.31 | 0.19 | 93 | 84.2 | 0.002*[a] |
| | Computer/mobile | 4 | 0.62 | 0.37–0.86 | 4.94 | <0.00001* | 74 | | |
| | Real-time feedback only | 1 | -0.03 | -0.30–0.24 | 0.22 | 0.83 | NA | | |
| Presence of instructor | Self-directed learning | 2 | 1.12 | -0.49–2.73 | 1.37 | 0.17 | 97 | 0 | 0.43 |
| | Instructor-guided | 6 | 0.45 | 0.13–0.78 | 2.73 | 0.006* | 84 | | |

a: significance for 1 trial only
