# Supplementary material for: The Effectiveness of Technology-Based Cardiopulmonary Resuscitation Training on the Skills and Knowledge of Adolescents: Systematic Review and Meta-analysis
Source: J Med Internet Res. 2022 Dec 15;24(12):e36423. doi: 10.2196/36423 (PMC9801268; doi:10.2196/36423)
Supplement: Multimedia Appendix 5 [file jmir_v24i12e36423_app5.pdf]

Subgroup analyses	No. of comparisons	Effect estimate	95%CI	Subgroup effect		I^2	Subgroup differences	
				Z	p-value		I^2	p-value
Subgroup analyses based on instructor guidance								
Self-directed learning	2	1.12	-0.49–2.73	1.37	0.17	97	0	0.43
Instructor-guided	6	0.45	0.13–0.78	2.73	0.006*	84		
Subgroup analyses based on hands-on practice								
Hands-on practice	6	0.45	0.13–0.78	2.73	0.006*	84	0	0.43
Without practical training	2	1.12	-0.49–2.73	1.37	0.17	97		
Subgroup analyses based on training modalities								
Video-instruction	3	0.80	-0.39–1.99	1.31	0.19	93	84.2	0.002*[a]
Computer/mobile	4	0.62	0.37–0.86	4.94	<0.00001*	74		
Real-time feedback only	1	-0.03	-0.30–0.24	0.22	0.83	NA		

a: significance due to 1 study only
